# Non-surgical Treatment of Severe Skeletal Class III Malocclusion in an Adolescent Patient: A Case Report

**DOI:** 10.7759/cureus.75796

**Published:** 2024-12-16

**Authors:** Radwan A Haffaf, Bilal F Mahmoud, Mohamad M Younes, Yarob A Ghanem, Mounzer Assad, Abdul-Kareem Hasan

**Affiliations:** 1 Department of Orthodontics, Tishreen University, Latakia, SYR; 2 Orthodontics, Ajyad Medical Center, Sharjah, ARE; 3 Department of Oral and Maxillofacial Surgery, Al-Wadi International University, Homs, SYR; 4 Department of Oral and Maxillofacial Surgery, Tishreen University, Latakia, SYR

**Keywords:** class iii malocclusion, non-surgical treatment, rapid maxillary expansion, rme, skeletal malocclusion

## Abstract

Class III malocclusion is one of the most challenging malocclusions in orthodontics. Its nature and multifactorial etiology should be taken into consideration, especially in growing and adolescent patients. Many treatment modalities have been proposed. The wide variety of underlying skeletal and dental components of this malocclusion justifies the different treatment options in the literature. The patient's decision, guided by the orthodontists and maxillofacial surgeons, is fundamental for choosing the appropriate treatment approach for this condition. A major component of this malocclusion is the maxillary transverse deficiency, which necessitates maxillary expansion. Rapid maxillary expansion (RME) has multiple advantages when incorporated into the treatment plan. RME is a type of maxillary expansion that separates the midpalatal suture using different appliances. This case report presents a borderline class III case where a non-surgical approach was adopted to treat an adolescent patient. The non-surgical approach was adopted based on the patient’s skeletal age, in addition to the patient's desire and decision. RME was involved followed by a fixed orthodontics appliance with class III elastics. The duration of treatment was 15 months. Forward maxillary movement was observed post-RME. An impacted upper second premolar erupted spontaneously after space gaining, anterior crossbite was corrected, and the ANB angle improved from -2.2 degrees to 1.3 degrees. Esthetically pleasant and functional occlusion was achieved.

## Introduction

Class III malocclusion represents one of the most challenging and multifactorial malocclusions in the field of orthodontics. The prevalence of class III malocclusion varies according to races, ethnic groups, and geographic distributions [1]. The multifactorial etiology of class III malocclusion includes heredity (which affects mandibular growth mainly), familiar genetics, and environmental factors (such as wrong postural habits, sucking habits, airway obstructions, tongue size, atypical swallowing, trauma, and other factors). The interaction between intrinsic and extrinsic etiological factors results in different expressions of this malocclusion [2-4]. Consequently, various combinations of skeletal and dentoalveolar disharmony may be associated with class III malocclusion [5]. Dental class III malocclusion is primarily classified according to the first permanent molars relationship, where the mandibular first permanent molar is positioned mesially in relation to the maxillary first permanent molar [2]. Skeletal class III discrepancy may arise from mandibular prognathism and maxillary retrognathism. This is due to the differential growth of the maxilla and the mandible in relation to each other. [3]. Different treatment modalities have been introduced based on the underlying causative factor of this malocclusion. Several factors should be considered such as growth pattern, skeletal maturity, the severity of sagittal discrepancy, dental compensations, the patient’s ability and tolerance for surgical intervention, and age [6].

Considering that this discrepancy is not self-corrected nor self-limited, early intervention through growth modification is paramount for the correction of this skeletal discrepancy during prepubertal stages. However, camouflage treatment and surgical interventions may be adopted in the later stages of adolescence and adulthood [7]. Class III malocclusion may be presented with a maxillary transverse deficiency in many cases, necessitating correction in the initial phases of treatment [7,8]. Skeletal maxillary protraction with rapid maxillary expansion (RME) has been proposed in the literature as a viable approach for the treatment of skeletal class III malocclusion [5,7,9-12]. However, it has been shown that RME has favorable effects on maxillary sagittal position. Point A moved forward following RME in multiple previous studies [13]. Notable, the maxillary changes and point A forward movement following RME have been documented in earlier research conducted by Biederman [14]. However, these spontaneous post-RME changes are still controversial and may not be clinically significant [15]. Furthermore, concerns have emerged regarding the long-term stability of results following early treatment of class III malocclusion [5].

The present case report describes a non-surgical treatment of skeletal class III malocclusion in an adolescent female patient with an unerupted maxillary second premolar, maxillary deficiency, and an anterior crossbite. The treatment plan includes RME followed by a comprehensive fixed orthodontic treatment.

## Case presentation

**Figure 1:**
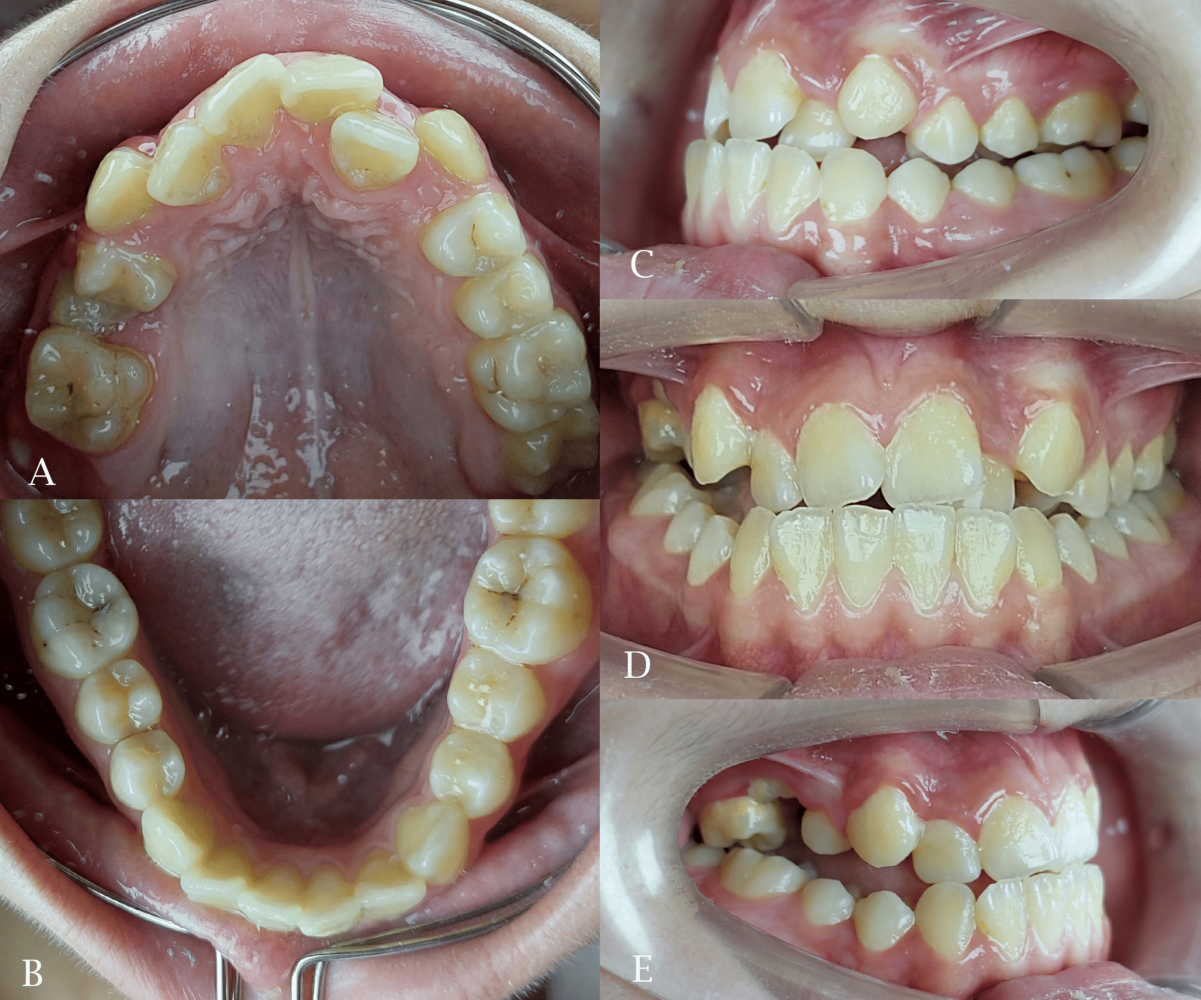
Pre-treatment intraoral photographs. A: maxillary occlusal view; B: mandibular occlusal view; C: left side view; D: frontal view; E: right side view

An adolescent female patient, aged 16 years, sought orthodontic treatment with the chief complaint of unpleasant smile. A thorough initial intraoral examination revealed a dental class III subdivision right, a palatally positioned left lateral incisor, and an anterior crossbite with a reverse overjet measuring -1.9 mm. Furthermore, the maxillary right second premolar wasn’t erupted and retained remnants of the primary second molar were observed. The upper and lower midlines were unmatched with blocked-out upper canines and right posterior open bite (Figure 1).

**Figure 2:**
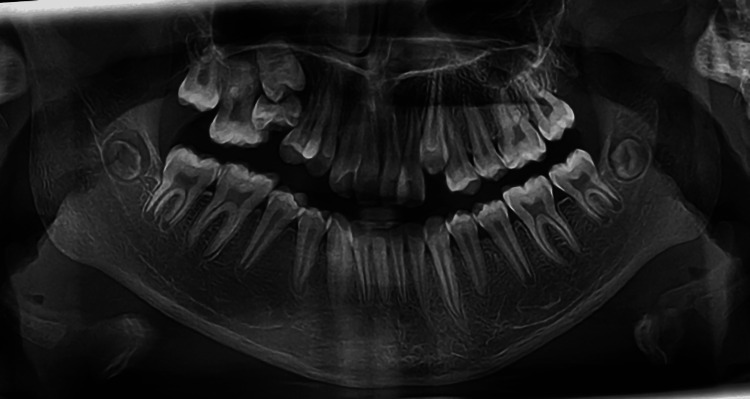
Pre-treatment panoramic radiograph.

The panoramic radiograph revealed that the second upper right premolar existed subgingivally, which exhibited mesial angulation and an altered position. The root of the premolar was not fully formed at this stage, suggesting the potential for spontaneous eruption if the retained remnants were extracted. Additionally, the adjacent molar was tilted mesially into the space of the premolar. The anterior nasal openings were nearly obstructed, which may indicate anterior upper airway constriction. This condition could be considered a contributing factor to the anterior maxillary constriction and transverse deficiency (Figure 2).

**Figure 3:**
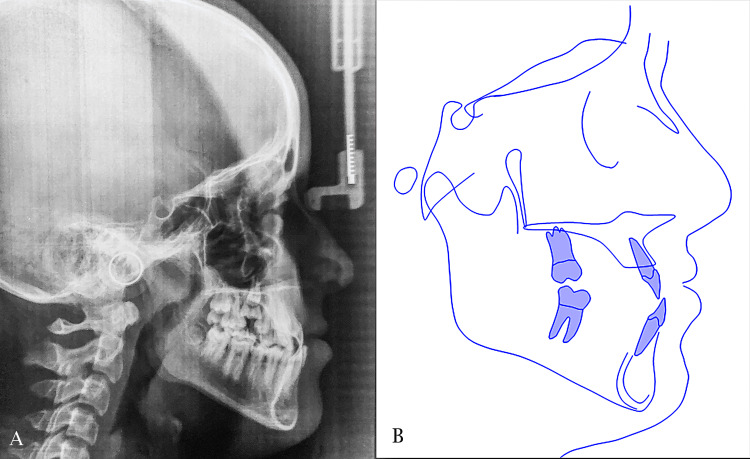
Pre-treatment lateral cephalometric evaluation. A: lateral cephalogram; B cephalometric tracing

Cephalometric analysis (Table 1) indicated a skeletal class III relationship (A point-Nasion-B point angle (ANB) = -2.2 degrees). Additionally, there was a tendency towered vertical height increase, as indicated by the lower anterior facial height to total anterior facial height (LAFH\TAFH) ratio of 56.6%. The angle formed between the mandibular plane and the sella turcica plane (SN-MP) measured at 39.2 degrees. The upper incisors were angulated normally relative to the maxillary skeletal base, with an angle of 110 degrees, as well as to the sella turcica line, which measured 98.1 degrees. The lower incisors were slightly proclined relative to the mandibular plane, measuring 93.7 degrees. Furthermore, both upper and lower lips were retruded in relation to the esthetic E line, with a measurement of -5.2 for the upper lip and -1.2 for the lower lip. A lateral cephalometric radiograph and tracing are presented in Figure 3.

**Table 1:** Pre-treatment cephalometric measurements. SNA: Sella point-Nasion-A point angle; SNB: Sella point-Nasion-B point angle; ANB: A point-Nasion-B point angle; SN-MP: the angle between mandibular plane (MP) and the sella turcica plane (SN); LAFH/TAFH: lower anterior facial height\total anterior facial height ratio; UI-SN: the angle between upper incisor axis (UI) and the sella turcica plane (SN); UI-MxP: the angle between the upper incisor axis (UI) and the maxillary plane (MxP); LI-MP: the angle between the lower incisor axis (LI) and the mandibular plane (MP); E - Upper Lip: the horizontal distance between the upper lip and the esthetic E line; E - Lower Lip: the horizontal distance between the lower lip and the esthetic E line

measurements	value
SNA	73.1°
SNB	75.3°
ANB	-2.2°
SN-MP	39.2°
LAFH/TAFH	56.6%
UI-SN	98.1°
UI-MxP	110.3°
LI-MP	93.7°
E - Upper Lip	-5.2 mm
E - Lower Lip	-1.2 mm
Overjet	-1.9 mm
Overbite	0.3 mm

Treatment plan

The treatment aims were correcting the sagittal discrepancy and the accompanying anterior crossbite, establishing normal overjet and overbite, facilitating the eruption of the maxillary second premolar, controlling the vertical dimension with minimal increase, and enhancing the aesthetic appearance of the smile while resolving the transverse deficiency.

Orthognathic surgery was proposed as a treatment option, but both the patient and her parents declined this option, opting instead for a non-surgical alternative. An informed consent was obtained following a comprehensive discussion regarding the advantages and disadvantages of each treatment option.

**Figure 4:**
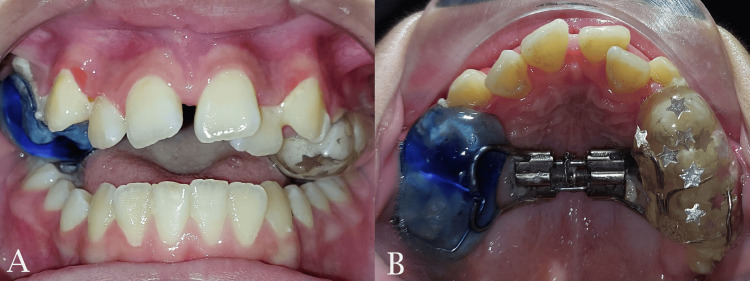
Separation of the midpalate suture and diastema appearance. A: frontal view; B: occlusal view

The patient’s skeletal maturity was assessed at stage CS4 according to the cervical vertebrae maturation (CVM) index. Despite the minimal likelihood of achieving midpalatal suture separation through conventional RME at this stage, the authors’ clinical experience indicated that the suture may not be fully fused in individuals of this age. RME was undertaken using the acrylic splint bonded expander. This type of expander incorporates posterior bite blocks which may prohibit any further vertical increase by utilizing masticatory forces in the posterior region. The activation protocol was three turns per day until the midline diastema and suture separation were observed (Figure 4), after which the protocol was adjusted to two turns per day until overcorrection in the posterior region was achieved.

**Figure 5:**
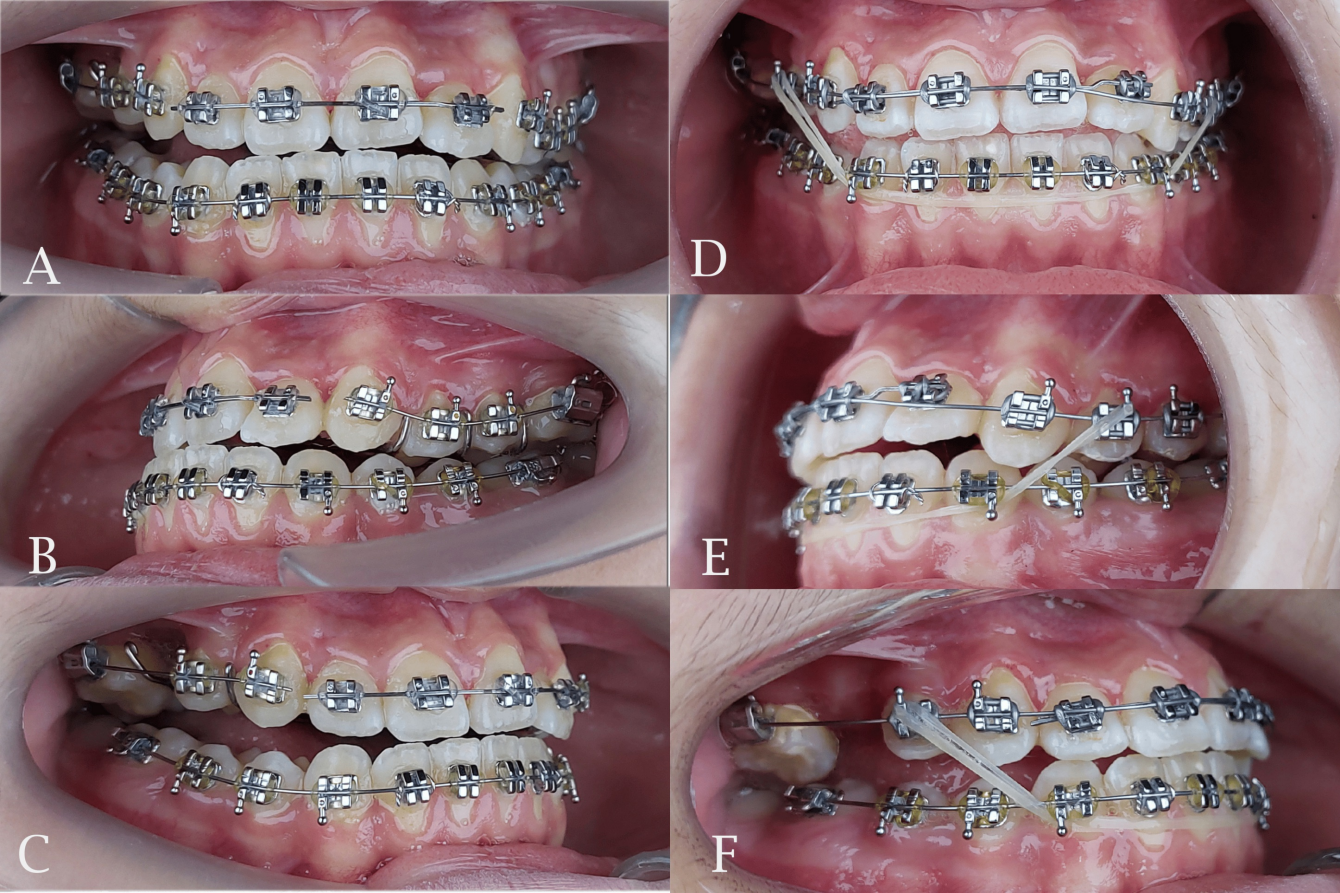
Initial fixed appliance bonding (left). Class III continuous elastics (right). A and D: frontal view; B and E: left side view; C and F: right side view

Following the initial phase of maxillary expansion, a comprehensive fixed orthodontic therapy was initiated. The treatment goals in the second phase were to further correct the sagittal discrepancy, and resolve all existing dental problems. The fixed orthodontic treatment was initiated with a 0.22 MBT bracket system. Upon achieving leveling and alignment of the maxillary dental arch, modified class III elastics (5\16 inch, 4.5 ounces) were employed as shown in Figure 5. Nickel-titanium (NiTi) open coils were utilized to facilitate space creation for the maxillary second premolar and to correct the angulation of the maxillary molar. Subsequently, a negative torque was applied using a stainless steel rectangular wire to adjust the inclination of the left lateral incisor.

It is noteworthy that during the leveling and alignment of the mandibular arch, only round archwires were utilized to avoid the complete expression of the bracket prescription, particularly in the anterior segment. This may prevent any exacerbation of the anterior crossbite and the reverse overjet.

Treatment results

**Figure 6:**
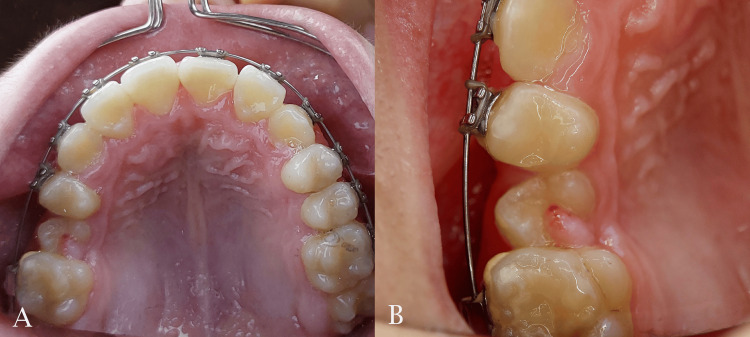
Spontaneous eruption of the maxillary premolar after space creation. A: maxillary occlusal view; B: close-up view of the premolar area

**Figure 7:**
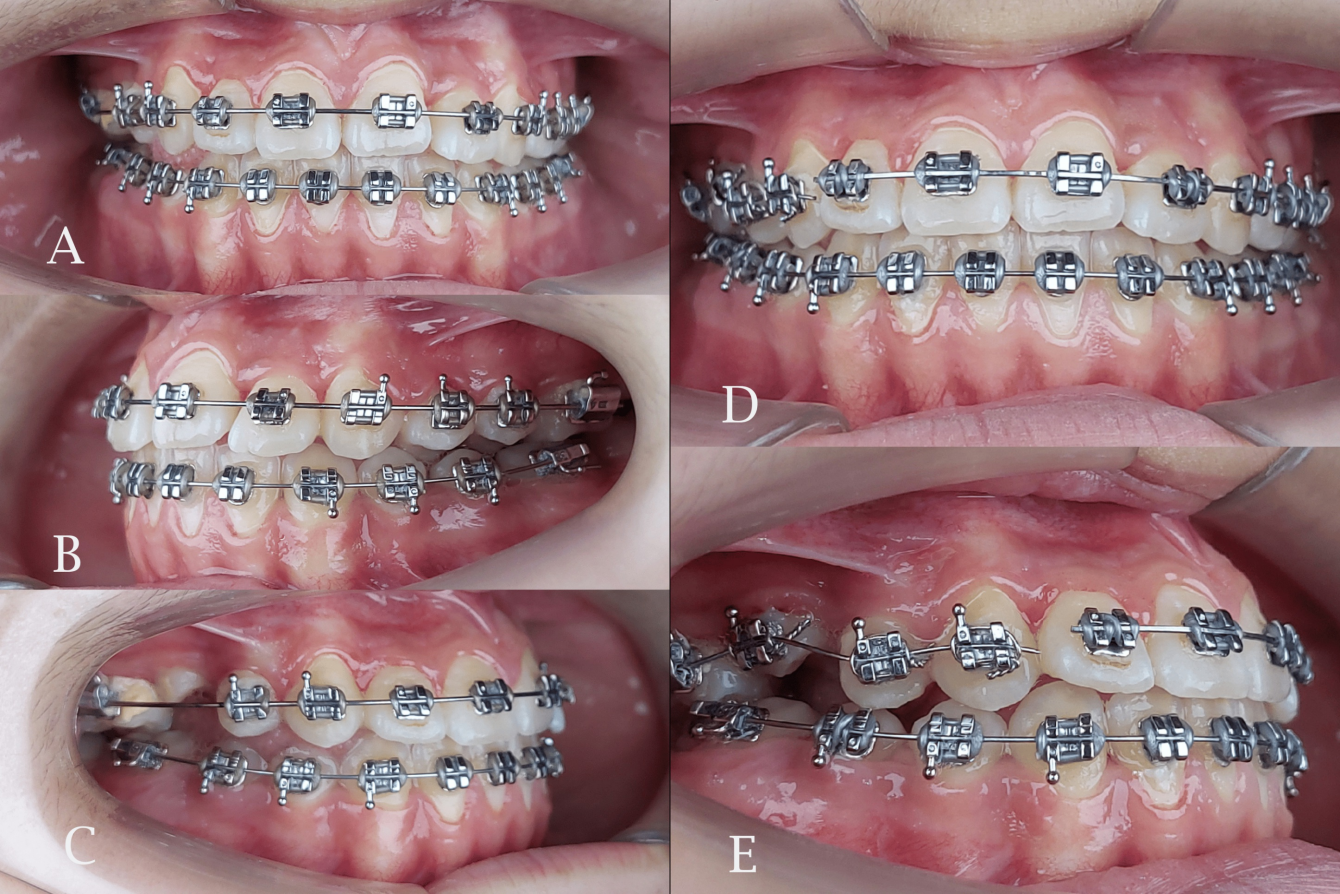
Sagittal correction after using class III elastics (left) and bonding the erupted second premolar (right). A and D: frontal view; B: left side view; C and E: right side view

**Figure 8:**
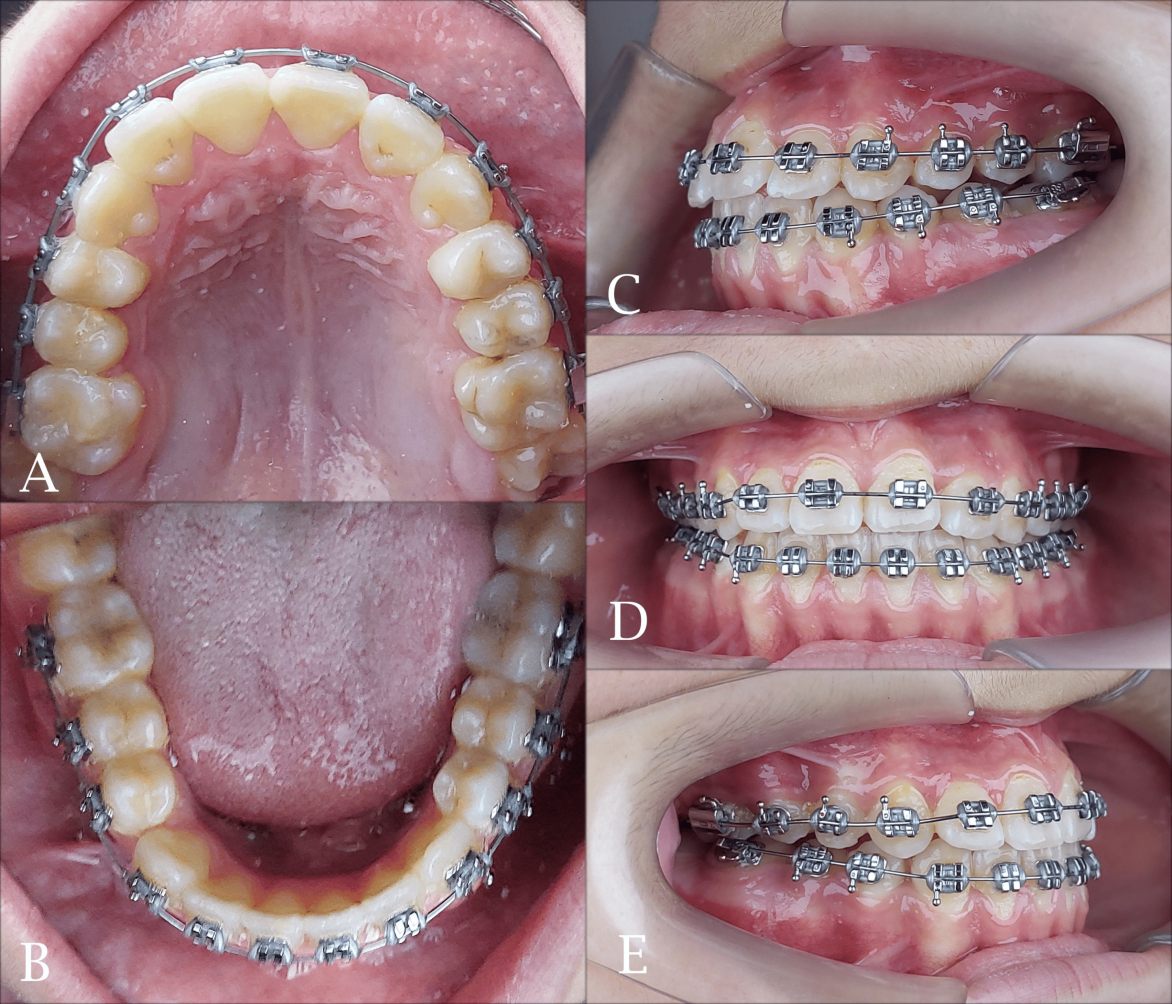
The final stage of fixed orthodontic treatment. A: maxillary occlusal view; B: mandibular occlusal view; C: left side view; D: frontal view; E: right side view

**Figure 9:**
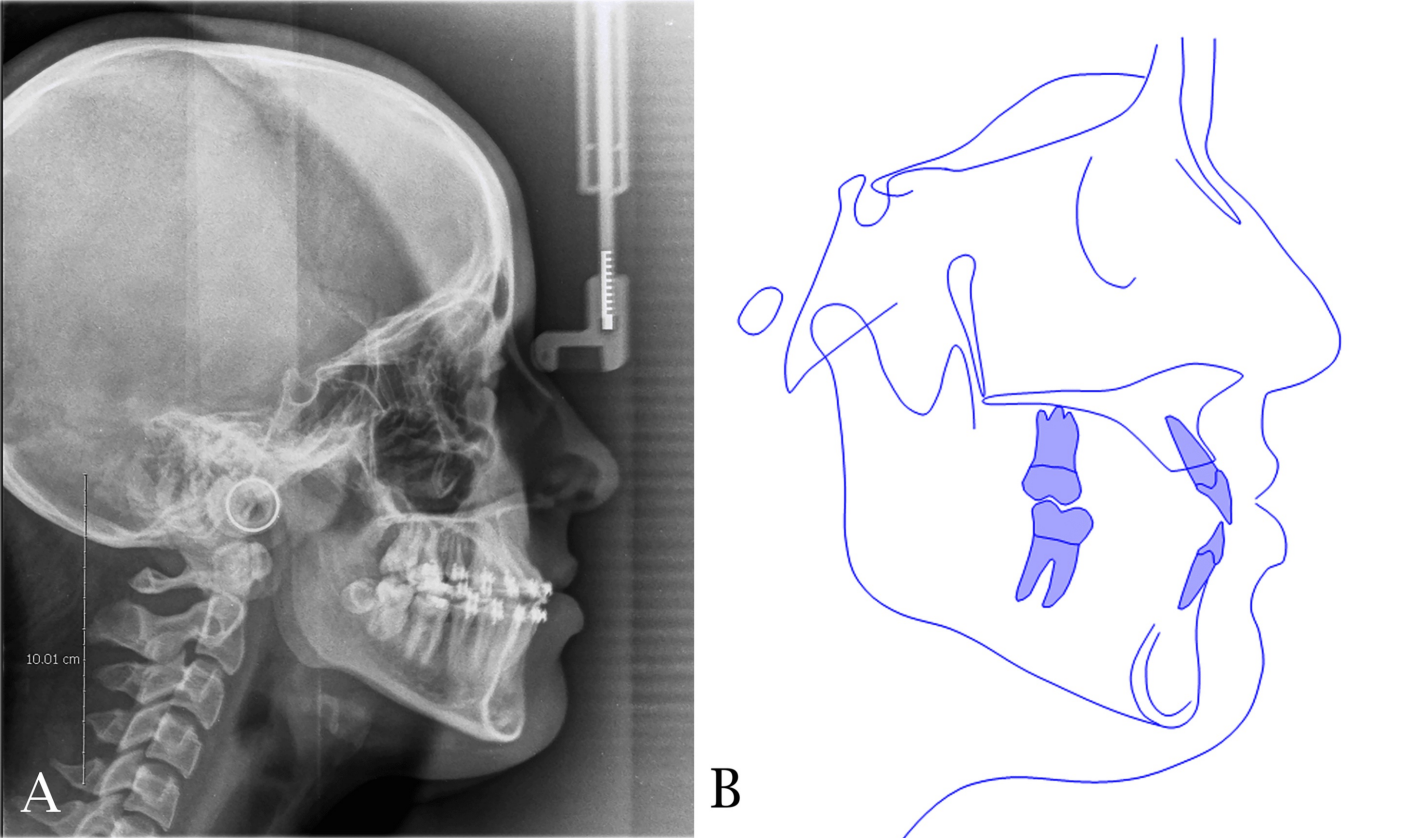
Pre-finishing lateral cephalometric evaluation. A: lateral cephalogram; B cephalometric tracing

**Figure 10:**
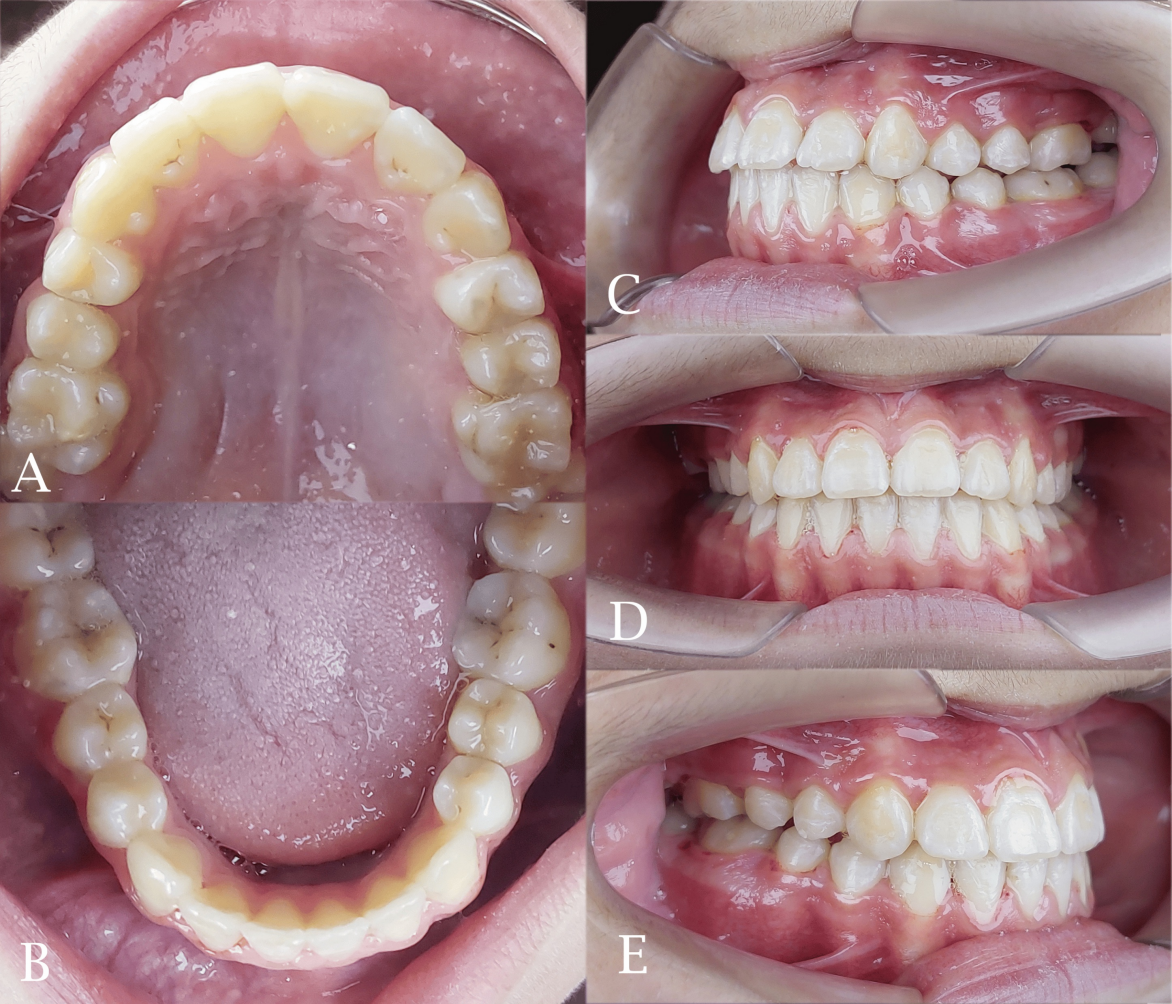
Post-treatment intraoral photographs. A: maxillary occlusal view; B: mandibular occlusal view; C: left side view; D: frontal view; E: right side view

**Figure 11:**
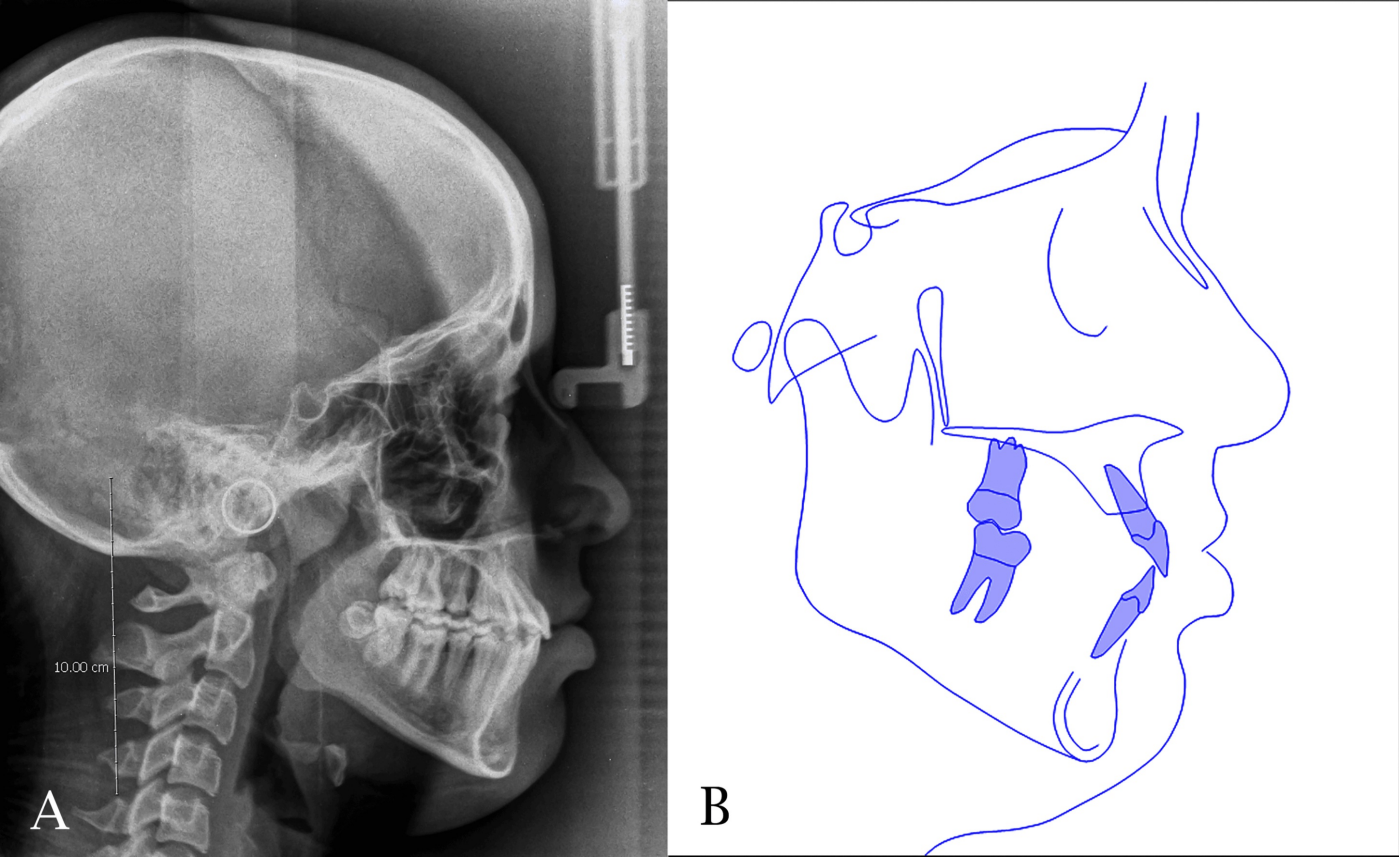
Post-treatment lateral cephalometric evaluation. A: lateral cephalogram; B cephalometric tracing

The midpalatal suture was successfully separated and this was confirmed by the appearance of midline diastema as shown previously. The resultant space created following maxillary expansion facilitated the alignment of the palatally positioned lateral incisor and the blocked-out canines. Sufficient space was gained to facilitate the eruption of the second premolar (Figures 6, 7). The relationships of canines and molars were class I with acceptable intercuspation (Figure 8). The cephalometric radiograph revealed axial correction of the tilted maxillary molar. The lower incisors maintained an acceptable labiolingual angulation (Figure 9). Notably, a reduction in the reverse overjet was observed following RME. Maxillary forward movement was confirmed through the lateral cephalometric analysis (Table 2). A complete correction of the anterior crossbite and the sagittal problem was achieved (Figure 10). The post-treatment lateral cephalometric evaluation is presented in Figure 11.

**Table 2:** Post-treatment cephalometric measurements. SNA: Sella point-Nasion-A point angle; SNB: Sella point-Nasion-B point angle; ANB: A point-Nasion-B point angle; SN-MP: the angle between mandibular plane (MP) and the sella turcica plane (SN); LAFH/TAFH: lower anterior facial height\total anterior facial height ratio; UI-SN: the angle between upper incisor axis (UI) and the sella turcica plane (SN); UI-MxP: the angle between the upper incisor axis (UI) and the maxillary plane (MxP); LI-MP: the angle between the lower incisor axis (LI) and the mandibular plane (MP); E - Upper Lip: the horizontal distance between the upper lip and the esthetic E line; E - Lower Lip: the horizontal distance between the lower lip and the esthetic E line

measurements	value
SNA	75.4°
SNB	74.2°
ANB	1.3°
SN-MP	40.6°
LAFH/TAFH	57.2 %
UI-SN	105.9°
UI-MxP	117.2°
LI-MP	92.5°
E - Upper Lip	-4.2 mm
E - Lower Lip	2.2 mm
Overjet	2.3 mm
Overbite	1.1 mm

## Discussion

The aim of the current case report was to demonstrate a non-surgical approach for the management of skeletal class III in an adolescent patient. It was a borderline case with severe transverse and sagittal deficiencies. The primary goals were to achieve a functional occlusion and acceptable aesthetics without surgical intervention. RME was utilized without an orthopedic face mask. The favorable effects of RME, along with the forward movement of the maxilla during the treatment, contributed to the correction of the sagittal discrepancy. Class III elastics were utilized to further address the sagittal discrepancy. Spontaneous eruption of maxillary right second premolar was observed following space gaining. The aesthetic outcomes were positively received by both the patient and her parents. The overall duration of treatment was 15 months.

The ANB angle changed significantly from -2.2 degrees prior to treatment to 1.3 degrees following treatment, indicating a positive correction of class III skeletal discrepancy. A forward movement of the maxilla was noticed, indicated by the SNA angle increases from 73.1 degrees prior to treatment to 74.2 degrees following treatment. The suggested mechanism for this spontaneous forward movement was previously explained by Biederman in the 1970s [14]. It was also shown by a study by Celebi et al. that RME may provide a spontaneous correction of an edge-to-edge incisor relationship and correction of the mild Class III relationship, even without a face mask [16]. Additionally, factors such as patient age (the maturity of the patient) may affect the location of the center of rotation during maxillary expansion, which in turn could affect the forward movement of the maxilla. The forward movement of point A is a result of the angular (wedge-shaped) displacement of the two halves of the maxilla, considering the fact that the anterior margin of the maxilla is free while the posterior margin is a continuous articulation [14,16]. However, the patient in this case report was assessed at stage 4 according to the cervical vertebrae maturation index (CVMS 4), yet forward maxillary movement was observed. A case report by Kuc-Michalska et al. showed that two of the four patients (the females) exhibited unexpected forward movement in the midface during post-pubertal stages, which was measured as an increase in the distance between condylon point and point A [5].

The SNB angle decreased from 75.3 degrees to 74.2 degrees. This reduction could be due to the minor vertical increase resulting from the palatal expansion which can induce a clockwise rotation of the mandible. The increase in the vertical dimension was acceptable, measuring at 0.6% (less than 1%). The combined effect of maxillary forward movement and mandibular clockwise rotation played a significant role in the partial correction of the sagittal discrepancy. The correction continued using class III elastics with various modifications. The overjet increased from -1.9 mm to 2.3 mm and the anterior crossbite was corrected completely.

Even though orthognathic surgery has the potential to transform the patient’s profile and enhance overall facial aesthetics, a non-surgical approach was chosen by many patients. This also reduces the complications associated with orthognathic surgery [17]. Implementing face mask therapy along with RME in the first stage was not selected, considering the patient’s classification at CS4, and a decision was made to continue with fixed orthodontics and utilize intermaxillary elastics. Previous studies have shown the effectiveness of these protocols at early age [9]. In adolescence, a case report by Pattanaik and Mishra [18] showed the effectiveness of face mask therapy in a similar patient. However, in a systematic review conducted by Raghupathy et al. [19], the authors assessed the stability following face mask therapy and they concluded that more focus should be given to restrict the mandibular movement in the long term.

The maxillary incisors were shown to be within the upper normal limit. The mechanics of class III treatment have the effect of protruding maxillary incisors and retruding mandibular incisors. The maxillary incisors were proclined by 7 degrees, while the mandibular were retroclined by 1.2 degrees. The mandibular incisors were in a preferred angular position relative to the mandibular plane following the treatment.

Vertical dimension was increased at the final stage as a result of utilizing maxillary expansion and class III elastics. Lineberger et al. [20] showed that RME can be used in patients with increased vertical dimension without detrimental effects. The LAFH/TAFH ratio increased by 0.6 % in the current case study, a change that is very acceptable given the complex mechanics employed for this patient.

The limitation of the current case report is that it used cephalometric evolution to interpret the outcomes of skeletal and dental movements. With the development of cone beam computed tomography (CBCT), clinicians can assess and monitor the facio-skeletal changes in a better way following treatment. However, CBCT was not used in the current case study to avoid unnecessary radiation exposure, considering that it would not provide any significant additional value to the patient.

## Conclusions

Non-surgical treatment in a borderline class III skeletal case was selected for a highly compliant patient. The outcomes showed a complete correction of both the class III malocclusion and the anterior crossbite. RME was utilized to address the transverse deficiency and resolving the dental crowding. RME has a positive effect regarding the treatment of class III malocclusion cases in adolescence and correcting the sagittal discrepancy. Combined with the effect of class III elastic, this approach may facilitate the management of borderline cases without surgical intervention.

The non-surgical approach successfully facilitated the treatment of an adolescent patient at the CS4 skeletal maturity stage. The final occlusion was functionally and aesthetically acceptable.
